# Influence of age and schooling in written discourse of healthy adults

**DOI:** 10.1186/s41155-020-00148-7

**Published:** 2020-06-08

**Authors:** Larissa Zanichelli, Rochele Paz Fonseca, Karin Zazo Ortiz

**Affiliations:** 1 Department of Speech, Language and Hearing Sciences, Universidade Federal de São Paulo (UNIFESP), Rua Botucatu 802, Sao Paulo, Brazil; 2 Universidade Católica do Rio Grande do Sul, Porto Alegre, Brazil

**Keywords:** Narrative discourse, Education, Schooling, Aging

## Abstract

**Background:**

Discourse production is a very complex cognitive task that requires the integration of several linguistic cognitive skills. Socio-demographic factors such as schooling can impact on cognitive tasks. This study investigated the impact of age and schooling in some macrolinguistic and microlinguistic aspects in the written discourse of healthy adults.

**Methods:**

Individuals with no previous history of language, hearing, neurological, or psychiatric disorders were asked to write a story based on a figure that showed a “bank robbery.” A total of 463 graphic narrative were analyzed. The schooling was stratified into the following three bands: 5 to 8 years, 9 to 11 years and 12 or more, and the age ranged from 19 to 75 years.

**Results:**

Individuals with high schooling (12 years or more) produced discourses with more information units, more coherent, and cohesive. The oldest group (60 to 75 years) needed more time to finish the written production.

**Conclusion:**

The schooling influences some micro and macrolinguistic aspects in the written discourse production. A higher educational level provided a greater number of words as well as a higher number of information units, and the discourses produced are more coherent and cohesive. The age influenced only the time of discourse production.

## Introduction

The impact of socio-demographic factors in cognitive tasks has been discussed due to the wide socio-cultural variety found in several countries, especially countries in development. It is known that age and schooling directly affect linguistic tasks such as naming by visual confrontation, dictation, oral comprehension of complex sentences, and reading sentences and texts (Soares & Ortiz, 2009; Pagliarin et al., 2014).

The education level is highly recognized as a factor that impacts in tasks that are used to assess not only the language, but also other cognitive functions, such as memory, attention, executive function, and even nonverbal cognitive tasks (Ardila, et al., 2010; Noronha, Barreto, & Ortiz, 2018). The discursive production is regarded as a complex task that requires the integration of several linguistic cognitive skills, such as memory, idea selection, organization, planning, and naming (Alexander, 2006; Rogalski et al., 2010; Andreetta, Cantagallo, & Marini, 2012; Wills, Capilouto, & Wright, 2012; Cannizzaro & Coelho, 2013). With respect to text production, planning and working memory are crucial processes in order to build a coherent text. Concerning the production of both oral and written texts, these processes stand out since all the content already stated is processed in order to produce them, as new information are sought and the idea is completed. Thus, it is a cyclical process that will be repeated numerous times throughout the text construction (Wills, Capilouto, & Wright, 2012). It is known that formal education develops these skills, in addition to promoting structural changes in the brain that provide better integration between the cerebral hemispheres and also increase the cognitive reserve.

At the same way, aging is a factor that can impact discourse production in several ways (Pistono, et al., 2017; Lira et al., 2018), and the effect of aging on the discourse has, until now, presented a confusing and ambiguous picture (Sherratt & Bryan, 2019). Some cognitive functions are influenced by age, and thus, they also impact the discourse production. With regard to cognitive changes in aging, it is possible to highlight the reduction of thinking speed, changes in the working memory, and in the visuospatial skills (Zanto et al., 2010). In addition, there may be a reduction in the information processing, as well as sensory and attention issues (Baddeley et al., 2011). There are also perceived changes in language (Freitas et al., 2006). According to the literature, some studies show differences in the production of narrative discourse by elderly, which may be considered less efficient, may require the use of extended pauses (longer time to carry out the task), may include irrelevant content and long narratives (Gaesser, Sacchetti, Addis, & Achacter, 2011; Saling et al., 2012; Miller, 2013), and there are increased cohesive errors (Babaei, Ghayoumi-Anaraki, & Mahmoodi-Bakhtiari, 2019).

Discourse can be defined as a form of language that goes beyond isolated sentences and a set of statements that are intended to convey a message between the interlocutors (Ulatowska & Olness, 2004). Three main aspects of linguistic processing can be focused in the discourse: microlinguistic or intra-sentence, which is responsible for phonological, lexical, and syntactic measures (intraphrasal and sentential functions); macrolinguistic or between sentences, which is responsible for functions between the sentences, considering local cohesion and overall coherence; and global processing of meaning, which is responsible for the formation of the mental model of a text (Marini et al., 2005).

There are several types of discourse: conversational, procedural, persuasive, expository, and narrative. Narratives can be considered one of the most common in communicative routine. Narratives include explanations of a scene, verbal reiterations of an event, spontaneous sharing of experiences, and stories–highly structured fiction forms (Wright et al., 2011). Narratives can be elicited in several ways, such as semi-directed interviews, spontaneous emissions, or from figures (Villiers & Villiers, 2010)**.** Discourse ability entails a complex interaction of linguistic, communicative, and other cognitive processes where a picture description task is considered the most effective way of obtaining a suitable discourse sample that can be standardized across many subjects. There are at least four benefits of using this instrument: (i) it provides a clear pictorial focus, thus reducing ambiguity about the subject matter; (ii) it reduces the demand on memory because the stimulus remains available to the subject at the time of evaluation; (iii) it minimizes confounders in analysis due to the controlled nature of the speech content; and (iv) when used to reevaluate, it monitors progression. (Duong & Ska, 2001; (Duong et al., 2005)**.** It is an important tool to elicit connected speech samples, and it can be really close to a natural conversation (Marini et al., 2005). Sampling of connected language is necessary to provide an accurate prediction of language competence in relevant contexts other than those in which the sampling occurs (McNeil, Doyle, Fossett, Park, & Goda, 2001).

The information units (IU) represent the amount of information provided by the subject. They are the informative and relevant elements that are present in an organized discourse structure. The successful discourse requires the combination of IU, as propositions, in a coherent way to convey a significant message (Wright, 2011). The IU are considered a sensitive measure to distinguish language disorders of different etiologies, as we can see between normal and aphasic adults, for example (Nicholas & Brookshire, 1995; Capilouto et al., 2005).

With respect to the production of written narrative, there is scarcity of studies on macrolinguistic and microlinguistic aspects analyzing age range and schooling level. The literature presents a predominance of studies that use schooling only as an inclusion/exclusion criterion, but in general, it is not used as a variable investigated. Regarding the oral narrative, previous studies described that schooling significantly impacted vocabulary indexes, content quality, as well as quality and clarity of referents (Juncos-Rabadan et al., 2005). There was also an effect of schooling on the size of the emission, that is, the number of words and content units (North et al., 1986).

Therefore, studies on narrative discourse in adults with written output and its relationship with the potential impact of age and schooling level are still necessary. According to a text processing model (Kintsch & Van Dijk, 1978), written textual production may require more planning time, and there is a greater possibility of formulating and reformulating ideas, when compared to oral narrative ones. In this sense, it is necessary to investigate precisely the variability of this production according to age and schooling in healthy individuals. The discourse analysis is seen as a sensitive tool in order to detect language disorders, and, indirectly, it also can be used to detect disorders in other domains of cognition (Duong et al., 2005; Lira et al., 2011). Therefore, the assumption of this paper is that age and schooling can directly impact individuals when performing this task, specially in a written form, since it is rarely investigated.

Understanding the structure of discourse is fundamental to the assessment and diagnosis of discourse level impairments in clinical distinct populations (Whitworth et al., 2015). Narrative discourse is a complex task involving the integration of information beyond the word level and requires individuals to remember concrete events and specific details, applying their knowledge of the world to construct a coherent structure of the narrative, where sequential order of events plays a key role (Diez-Itza, Martínez, & Antón, 2016). How aging and schooling can interfere on the construction of mental models and on implicit structure to the way in which adults will organize and write their discourse still needs clarification.

Thus, the purpose of this study was to analyze the impact of age and schooling level on some macrolinguistic and microlinguistic aspects of the written discourse of healthy adults, once it is known that socio-demographic factors can impact in language tasks, in order to check specific aspects of this interference on the written narrative production.

## Methods

This is a retrospective study which was conducted at the Department of Speech, Language, and Hearing Sciences at the Universidade Federal de São Paulo. Data were analyzed from a previous study approved by the Research Ethics Committee of the Universidade Federal de São Paulo (No. 2414/08) and by Pontifícia Universidade Católica do Rio Grande do Sul (No. 04908). After receiving full information about the study, written informed consent was obtained from all enrolled.

In a previous study, the sample was composed by 500 healthy volunteers age ranged from 19 to 75 years, with no previous history of neurologic or psychiatric diseases, uncontrolled systemic diseases, self-reported communication disturbances, complaints of cognitive difficulties, use of psychotrophic medication, history of alcohol abuse or use of illegal drugs, uncorrected visual, or auditory deficits that could affect test performance. This information was collected by applying a questionnaire.

At first, the individuals were submitted to screening cognitive tasks: Mini Mental State Examination (MMSE) and Clock Drawing Test (CDT) (Folstein, Folstein, & Mchugh, 1975; Juby et al, 2002; Critchley, 1953). The MMSE included the population criteria according to the educational level (Brucki, et al., 2003): − 20 points to illiterate people, 25 points to people with 1 to 4 years of education, 26. 5 to people with 5 to 8 years of education, 28 for those with 9 to 11 years of education, and 29 for those with more than 11 years of education). For the CDT, scores were analyzed assuming > 7 as cutting point for cognitive screening (Sunderland et al., 1989). According to a previous study, the association of both tests was considered a good procedure for cognitive screening (Juby, Tench, & Baker, 2002). All participants that met the inclusion criteria were submitted to the written discourse task.

A total of 500 healthy adults took part in this study. Of this group, 37 have refused to write a history. Thus, 463 discourses were analyzed. In this way, 463 discourses were analyzed. In this task, the subjects were asked to write a story based on a figure: “Bank Robbery.”

The individual was instructed as follows: “Do you see this picture? I would like you to write me the story described here. You may look as long as you think necessary before beginning.” The test began the moment the subject started to write the tale and terminated only when he/she indicated that there was nothing more to be written. Next, the evaluator asked the subject whether he/she had anything more to add. For those subjects who had difficulty beginning the narration, the evaluator encouraged them without influencing the individual’s written production. The test was performed individually in a quiet room.

### Data analysis

All discourses were initially analyzed using *The Vantage Point* software. The software uses a word counting mechanism which is able to list all the different words used in discourses, and then it combines semantically related words to indicate how many times a particular word or semantically related terms were included in the discourses. Therefore, this analysis was used to identify the ten open class words that were included most often in the discourses analyzed. These included bakery, assault and/or robbery and/or steal, mugger, police and/or security guard and/or police officer, truck and/or van, run, wait, warn/call, person, and gun. These words are consider the IU. In this way, after the identification the most important information units, all discourses were analyzed again, now according to the interest variables: macrolinguistic and microlinguistic aspects for subsequently check whether the age and education influence these aspects. The following microlinguistic aspects were analyzed: total number of words and total (IU) produced, and 1 point was awarded for each word and for each IU included. In addition, the total production time was measured. Another aspect analyzed was the presence of the essential elements of the scene/figure. The “Bank Robbery” consists of 3 elements: (1) the assault inside the agency, (2) someone outside waiting for the thieves, and (3) someone calling/warning the police. One point was awarded to each element. The subject could reach a maximum of 3 points in this part of the task.

The macrolinguistic aspects of the discourse were also analyzed: cohesion and coherence. One point was given to the cohesion if the discourse represented a sequence compatible with the scene, and 1 point was given to coherence if the subject produced the discourse only with content related to the theme of the figure. From these aspects, it was possible to classify the discourse as a “narration” or just as a “description of the scene.”

To check the impact of the age and schooling in aspects of the discourse, the individuals were gathered in five age groups: 19 to 29 years, 30 to 39 years, 40 to 49 years, 50 to 59 years, and 60 to 75 years old; and in three educational levels: people with 5 to 8 years of education, people with 8 to 12 years of education, and those with more than 12 years of education. In this study, we chose to not include individuals with low educational level (1 to 4 years of study), as there are many functional illiterates in this range, that is, a person who, despite having attended the school did not develop the reading and writing skills that are required to personal and professional development (Eme et al., 2010).

### Statistical analysis

In order to evaluate the influence of demographic data (gender, age, and education) in the elements of the discourse, regression analyses were conducted in which the elements of the discourse were dependent variables and demographic data were independent variables. As reference groups, we used females, from 19 to 39 years and with 5 to 8 years of study. Logistic regressions were conducted if the dependent variable was categorical. The results were presented on the odds ratio (OR). Linear regression analyzes were performed when the dependent variables were discrete or continuous, presenting the results of the β coefficients. There were no missing data.

The probability (*p*) under 0.05 was considered as an indication of statistical significance. All tests were two-tailed. A ninety-five percent confidence interval (CI) was calculated in relation to the β coefficient and the OR. The whole analysis was calculated in accordance with the STATA version 12 statistical package.

## Results

### General characteristics

The age of the 463 participants ranged from 19 to 75 years (mean ± SD 44. 8 ± 15.1) and 66% of the participants were women. The years of education ranged from 5 to 25 years (average ± SD 11. 2 ± 4.4).

### General characteristics of written discourse

The number of words ranged from 3 to 112 (median was 36), and the time taken to produce the discourse ranged from 15 to 560 s (median was 122 s). Considering the scene, 33% were descriptions, and 67% were narratives. The median of the IU total score was 7 (ranging from 0 to 10). The median of the elements total score was 2 (ranging from 0 to 3). In relation to macrolinguistic aspects of the discourse, 94% of the participants presented a cohesive discourse, and 94% presented a coherent one.

### Relationship between written discourse, gender, age, and educational level

It is notable that age impacted the time of discursive production, as older individuals spent more time to write the story. In relation to age, only the group from 40 to 59 years provided more descriptions than narrations when compared to other groups (Tables 1 and 2). Most male participants described the scene, rather than narrate it (Tables 1 and 2). Overall, the schooling level influenced all elements of the discourse (Tables 1 and 2).

**Table 1:** Analysis by linear regression to evaluate the influence of demographic data in discourse

Speech elements	***β***	Standard error	***p***	CI 95% (***β***)
No. of words				
Males	− 0.5	1.6	0.771	− 3.7; 2.7
Age range, 40–59	0.2	1.7	0.906	− 3.1; 3.5
Age range, 60–75	− 1.1	2.1	0.582	− 5.2; 3.0
Education level, 9–11	6.8	1.8	< 0.001	3.2; 10.4
Education level:,≥ 12	20.1	1.8	< 0.001	16.5; 23.7
Production time				
Males	− 1.8	7.2	0.801	− 15.9; 12.3
Age range, 40–59	7.9	7.5	0.291	− 6.8; 22.7
Age range, 60–75	23.8	9.3	0.011	5.6; 42.0
Education level, 9–11	10.2	8.1	0.212	− 5.8; 26.2
Education level, ≥ 12	36.1	8.1	< 0.001	20.1; 52.0
Number of IU				
Males	0.1	0.2	0.550	− 0.3; 0.5
Age range, 40–59	− 0.1	0.2	0.777	− 0.5; 0.4
Age range, 60–75	0.0	0.3	0.955	− 0.5; 0.5
Education level, 9–11	1.3	0.2	< 0.001	0.9; 1.8
Education level, ≥ 12	2.3	0.2	< 0.001	1.9; 2.8
Number of elements				
Males	0.0	0.1	0.784	− 0.2; 0.1
Age range, 40–59	0.0	0.1	0.803	− 0.2; 0.1
Age range, 60–75	− 0.1	0.1	0.313	− 0.3; 0.1
Education level, 9–11	0.5	0.1	< 0.001	0.3; 0.7
Education level, ≥ 12	0.8	0.1	< 0.001	0.7; 1.0

Age in years, reference group, 19–39 years

Education level in years, reference group, 5–8 years

**Table 2:** Analysis by logistic regression to evaluate the influence of demographic data in discourse

Speech elements	OR	Standard error	***p***	CI 95% (OR)
Scene description				
Males	2.0	0.4	0.001	1.3; 3.1
Age range, 40–59	1.6	0.4	0.052	1.0; 2.5
Age range, 60–75	1.6	0.5	0.082	0.9; 2.8
Education level, 9–11	0.7	0.2	0.166	0.4; 1.1
Education level, ≥ 12	0.6	0.1	0.018	0.3; 0.9
Scene narration				
Males	0.5	0.1	0.002	0.3; 0.8
Age range, 40–59	0.6	0.1	0.033	0.4; 1.0
Age range, 60–75	0.6	0.2	0.059	0.3; 1.0
Education level, 9–11	1.4	0.3	0.203	0.8; 2.2
Education level, ≥ 12	1.8	0.4	0.018	1.1; 2.9
Discourse coherence				
Males	2.8	1.6	0.070	0.9; 8.5
Age range, 40–59	0.8	0.4	0.680	0.3; 2.1
Age range, 60–75	0.7	0.4	0.526	0.2; 2.1
Education level, 9–11	2.6	1.2	0.040	1.0; 6.5
Education level, ≥ 12	9.4	7.1	0.003	2.1; 41.4
Discourse cohesion				
Males	0.7	0.3	0.466	0.3; 1.7
Age range, 40–59	0.7	0.3	0.466	0.3; 1.7
Age range, 60–75	0.9	0.5	0.905	0.3; 3.0
Education level, 9–11	2.9	1.3	0.021	1.2; 7.1
Education level, ≥ 12	10.6	8.0	0.002	2.4; 46.4

Age in years, reference group, 19–39 years

Education level in years, reference group 5–8 years

## Discussion

The most important findings of this study were that both factors—age and schooling—influenced the macrolinguistic and microlinguistic aspects of the written discourse, while the schooling level was the most preponderant factor. These findings are discussed below.

No similar studies, in which the variable number of words, IUs, textual cohesion, and coherence and the time, had been investigated in written discourse production. In general, most studies are done with oral discourses. In these studies, it could be found that the higher the education level, the better the performance of the individual in cognitive tasks, including narrative production (Mougias et al., 2019; Lyketsos et al., 1999). In a few studies with written production, the impact of schooling on narratives was observed specially when it was associated with reading and writing habits that improved the performance (Pagliarin, et al., 2015). The authors also found that schooling interfered differently in oral and written narratives, since written narratives require reading-writing abilities that are developed over the course of education. However, education was not a predictor of the tasks relating to oral narrative task (total number of words). In fact, tasks involving graphic stimuli (written comprehension, dictation, reading, written naming, number dictation, reading of numbers, written narrative, and written text comprehension) tend to be more sensitive to the influence of education (Ortiz, & Costa, 2011). The impact of schooling on written narratives could also be found even when comparing low educational level with a very low educational level (Akashi, & Ortiz, 2018).

Regarding to the number of IUs, it was possible to notice that the number of IUs increases with higher educational levels. The IUs previously set are important elements for the narration and compose the microlinguistic aspects of the discourse. Therefore, it is understood that the greater the number of IUs in the discourse, the greater the representativeness of the story provided. The proper development of the lexical and semantic aspects is necessary in order to be able to properly select the IUs. The findings of this study corroborate with the literature that indicates that there is an impact of schooling on the number of content units (semantic aspect) previously noticed for figures description and naming tasks (Le Dorze & Bedard, 1998; Mackenzie, 2000). Other cognitive aspects can also interfere with the discourse production. The complexity of this task implies in the development of skills, such as focused concentrated attention, planning, and working memory. These skills are best developed in individuals with higher educational level.

In the group with 12 years or more years of study, it was possible to notice that more words were used, and a longer production time was required to produce the written narrative. These individuals produced discourses with more words and IU, and then it took longer to complete the task. These findings corroborate with studies that showed an increase content and a greater number of words per minute in individuals with higher education level (Le Dorze & Bedard, 1998; Mackenzie, 2000). It is known that the literacy/education provides the development of linguistic cognitive skills, such as grammatical competence, vocabulary, working memory, attention and planning, what enables a discourse production with more complete syntactic and grammatical structures, and it allows larger narratives (Lira et al., 2011.

With regard to the macrolinguistic aspects of the discourse, as the group with the highest education level (12 years or more) presented a maximum score, it was possible to notice that the higher the education level, the greater the number of individuals who produced a narrative with coherence and cohesion. The “coherence” term has a difficult constitutive and operational definition (Stemmer & Whitaker, 2008). Global coherence is a macrolinguistic measure of the higher-level conceptual maintenance of topic across the discourse as a whole, while local coherence is the continued maintenance of content from one utterance to the next (Glosser and Deser, 1991). It is also known that coherence has a relationship of dependency with the cohesive elements, such as the connectives, which allow the continuity of the text, and establish relationships between parts of the discourse. Coherence and cohesion are linguistic phenomena that can be developed, while schooling, more specifically the acquisition of reading and writing, is a decisive factor in this progression (Cieri, 1985). Studies that investigated the influence of schooling on narrative production showed that the most complex, cohesive, and coherence texts were produced by individuals with higher schooling levels. It is due to the fact that, during the process of learning, the subject is exposed to many opportunities of textual production which would provide greater experience to prepare the written narrative (Spinillo & Pinto, 1994), and also coherence refers to a cognitive representation between linguistic/discourse characteristics and world knowledge (Babaei et al., 2019) influenced by schooling.

The performance of individuals on this item is related to the presence of the three scenes addressed in figure used as target: the heist, someone outside waiting for the thieves, and someone warning the police. Comparing the group with higher education level (12 years or more) with the group with lower education level (5 to 8 years), it was found that the first presented more scenes in their written discourse when compared to the second group. It is understood that the coherence of a written narrative discourse is directly linked to its content and to the typical structural components of a particular text genre, such as the description of the scene and characters, chain of events/plots, outcome, and resolution of the story (Wills et al., 2012). Studies still emphasize the relevance of inserting structural elements into causal relationships in narrative discourse, since it seems that the coherence of discourse will be impaired if these elements are not present (Bobrow & Collins, 1975; Juncos-Rabadan et al., 2005).Therefore, the discourses that presented the three elements of the scene were also more consistent, since they presented the structural components related to the narrative production. Another aspect to be raised is that the discourse was elicited from a visual information and that schooling can impact the performance of visual inferences.

It is known that the discourse is a complex task that involves retrieving information from memory, deciding on which elements should be included or excluded, and considering what the listener may or may not know and, as so, remain on the subject over time (Brownell & Joanette, 1993). The literature shows a strong correlation between cognition and macrolinguistic aspects of discourse, whereas this involves memory, planning, and attention which has important role in the maintenance of the topic during the production (Rogalski et al., 2010; Alexander, 2006). Discourse involves ongoing interactions among diverse cognitive processes including semantic storage and retrieval, executive functions and working memory (Mueller and Turkstra, 2018), and these cognitive processes are mentioned as key components for maintenance of a coherent discourse (Baddeley, 1986).

The hypothesis that cognition may be related to the discourse processing and also to education was considered due to studies that found that individuals who produced more consistent discourses obtained good performance on cognitive tasks and presented a higher level of education (Rogalski et al., 2010.

Regarding to age, it was found that there was statistically difference between the group of young people and the group of elderly people only to the time of written production. This finding differs from other studies that have found that ageing significantly increased the number of cohesive errors and reduced the quantity of the referential ties in picture-sequence narratives (Sherratt & Bryan, 2019) and decreased discourse ability for cohesion and coherence (Babaei et al., 2019). Many factors could explain the differences between our and previous studies such as type (genre) of the discourse, our elderly group with subjects up to 75 years, and the stimulus (Bank Robbery picture) that apparently is easier than those described in other studies.

Related to age, the main finding was that the higher the age, the greater the time to complete the task. In this study, although elderly group spent more time to write the story, no increase was observed in the number of words. The literature shows that when analyzing the narrative discourse, it is possible to find greater difficulty in word finding (Santos Nogueira, Azevedo Reis, & Vieira, 2016) memory and attention deficit and greater number of breaks, thus requiring more time to the individual to provide all the necessary information and the content (Marini et al., 2005; Le Dorze and Bedard, 1998; Mougias et al., 2019).

The type of stimulus that elicited the narrative production could also have interfered on the results. Some authors studied the influence of the type of stimulus that elicits the discourse in healthy individuals (Marini et al., 2005; Duong & Ska, 2001). Apparently, there is a difference between presenting a unique character or a sequence of figures. When the narrative production from a unique character with a sequence of figures was compared in these studies, they noted that despite the sequence of figures represent a facilitator for discourse production in elderly individuals and low schooling level, there was a greater number of words, use of undefined words, smaller number of main ideas, and repetition of information. As previously mentioned, the unique figure used in this study could have provided a greater number of descriptive discourses, instead of narrative discourses, in the elderly.

In this study, we could observe that written discourse is also influenced by schooling and age as pointed out by studies with oral/spoken discourses and other cognitive tasks. Although that, it does not mean that socio-demographic factors modify oral and written discourses in the same way. Besides, our data showed that it is possible to assess/analyze written narratives in individuals with at least 5 years of schooling. The possibility of the written analysis is very important considering different populations in which written and oral discourse are both necessary for language assessment. It is known that in specific language disorders, such as aphasia, i.e., oral and written language can be affected in different ways. So, data related to the written discourse are very important for a complete language assessment, and more data from normal populations are necessary for this comparision during the assessment. In this research field, it is also important to considerate that spoken and written discourse production might be not exactly the same even considering healthy subjects.

## Limitations of this study

The present study analyzed the number of words, the IU, cohesion and coherence, and time spent during written discourse production considering schooling and age. This should be interpreted in the light of some limitations. Many other discourse elements, such as lexical and syntatic aspects, content, propositions, global coherence, and macropropositions need further investigations. Studies that control age and schooling and compare oral and written discourses could also be helpful, considering that most studies are done with oral production. These studies could help to understand if (and how) years of schooling interfere on mental narrative schema and if written production reinforces it. In addition, the elderly population assessed in this study was not very very old which may have influenced the results. Future studies should investigate the discourse aspects that were not investigated in the present study and evaluate very very old people for group comparisions related to age.

## Conclusion

The schooling influenced some micro and macrolinguistic aspects in the written discourse. A higher schooling level provided a greater number of words as well as a higher number of IUs, and discourses produced are more coherent and cohesive.

This study reinforces the need of establishing clinical standards distinguishing age and schooling during the evaluation of the written discourse production. The performance of individuals with language disorders should be analyzed taking into account the schooling level and age factors, since socio-demographic factors directly impact the performance of healthy individuals in the written narrative discourse task.

## Data Availability

The materials are published, and the data are available.

## Abbreviations

IU: Information unit
MMSE: Mini Mental State Examination
CDT: Clock Drawing Test
OR: Odd ratio
CI: Confidence interval
